# A comparative study of the ability of recombinant oncolytic adenovirus, doxorubicin and tamoxifen to inhibit the proliferation of breast cancer cells

**DOI:** 10.1111/jcmm.17549

**Published:** 2022-09-23

**Authors:** Shanzhi Li, Zhuoxin Li, Yiquan Li, Yilong Zhu, Jicheng Han, Wenjie Li, Ningyi Jin, Jinbo Fang, Xiao Li, Guangze Zhu

**Affiliations:** ^1^ Academiciann Workstation of Jilin Province Changchun University of Chinese Medicine Changchun China; ^2^ Changchun Veterinary Research Institute Chinese Academy of Agricultural Sciences Changchun China; ^3^ College of Animal Science and Technology Jilin Agricultural University Changchun China

**Keywords:** apoptosis, breast cancer, chemotherapy drugs, luciferase, oncolytic virus

## Abstract

In this study, we compared the inhibitory effects of recombinant oncolytic adenovirus (Ad‐apoptin‐hTERTp‐E1a, Ad‐VT) with that of doxorubicin (DOX), a first‐line chemotherapy drug, and tamoxifen (TAM), an endocrine therapy drug, on the proliferation of breast cancer cells. We found that Ad‐VT could effectively inhibit the proliferation of breast cancer cells (*p* < 0.01); the inhibition rate of Ad‐VT on normal mammary epithelial MCF‐10A cells was less than 20%. DOX can effectively inhibit the proliferation of breast cancer cells and also has a strong inhibitory effect on MCF‐10A cells (*p* < 0.01). TAM also has a strong inhibitory effect on breast cancer cells, among which the oestrogen‐dependent MCF‐7 cell inhibition was stronger (*p* < 0.01), At higher concentrations, TAM also had a high rate of inhibition (>70%) on the proliferation of MCF‐10A cells. We also found that both recombinant adenovirus and both drugs could successfully induce tumour cell apoptosis. Further Western blot results showed that the recombinant adenovirus killed breast cancer cells through the endogenous apoptotic pathway. Analysis of the nude mouse subcutaneous breast cancer model showed that Ad‐VT significantly inhibited tumour growth (the luminescence rate of cancer cells was reduced by more than 90%) and improved the survival rate of tumour‐bearing mice (*p* < 0.01). Compared with DOX and TAM, Ad‐VT has a significant inhibitory effect on breast cancer cells, but almost no inhibitory effect on normal breast epithelial cells, and this inhibitory effect is mainly through the endogenous apoptotic pathway. These results indicate that Ad‐VT has significant potential as a drug for the treatment of breast cancer.

## 1 INTRODUCTION

The incidence of breast cancer has increased rapidly worldwide over the last few years^1^, ^2^ and now ranks first as the most common form of cancer in women.^3^

At present, the most common treatment for breast cancer is still the combination of surgical treatment and chemotherapy.^4^, ^5^ Doxorubicin is the main chemotherapy drug in clinical treatment of breast cancer. Adjuvant radiotherapy, chemotherapy, and endocrine and HER2‐guided therapy are all clinical strategies that can significantly reduce the risk of disease recurrence and improve the overall survival rate of patients with breast cancer. However, the non‐targeted toxicity of chemotherapy can seriously affect the quality of life of patients and approximately 40% of patients will relapse and die from metastasis.^3^ In addition to chemotherapy and radiotherapy, the endocrine drug tamoxifen has become an indispensable clinical treatment for breast cancer. Although this drug has fewer side effects, it also has significant limitations. Therefore, there is an urgent need to find a drug with a strong inhibitory effect on the growth of various breast cancers.

The clinical concept of using a virus to treat cancer began in the mid‐twentieth century. However, it was not until the 1990s that the emergence of recombinant DNA technology and viral genome engineering triggered a new wave of viral therapy. Viral therapy is a form of cancer treatment that uses viruses as vectors in which genetic engineering is used to modify the viruses themselves so that they are more inclined to target cancer cells; these viruses also alert the host's immune system to the presence of cancer. Viral therapy can be divided into two main types: (1) the use of non‐replicating viruses as vectors for tumour gene therapy and (2) the use of replicating viruses as oncolytic agents. The emergence of oncolytic adenoviral therapy has led to a significant reduction in the side effects of chemotherapy.^4^

There are many types of cell death, mainly divided into programmed cell death (apoptosis, necroptosis, pyroptosis, ferroptosis and autophagy) and cell unprogrammed necrosis. Of these, apoptosis is the most widely studied. Identifying ways to increase apoptosis and subsequent death in cancer cell death, thus inhibiting the progression of cancer, could provide a new concept for the treatment of cancer. Apoptin is a protein that is derived from the chicken anaemia virus and can selectively kill a variety of cancer cells.^6^ In tumour cells, apoptin undergoes phosphorylation in the nucleus; however, in normal cells, this process occurs in the cytoplasm.^7^

The transcription of human telomerase reverse transcriptase is a major step in the regulation of telomerase activity. Telomerase activity is essential for cancer cells to maintain immortality. By interfering with telomerase activity, it is possible to inhibit the growth of cancer cells.

**FIGURE 1:**
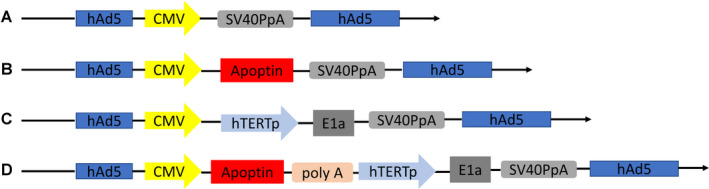
Schematic diagram of four recombinant adenovirus vectors constructed using shuttle vectors in our laboratory: (A) MOCK, (B) Ad‐Apoptin (Ad‐VP3), (C) Ad‐hTERTp‐E1a (Ad‐T) and (D) Ad‐Apoptin‐hTERTp‐E1a (Ad‐VT)

The recombinant adenoviruses (Ad‐VT, Ad‐T, Ad‐VP3 and Mock) constructed by our group are based on the RAPAd.I packaging system (Figure 1). Ad‐VT (Ad‐Apoptin‐hTERTp‐E1A) includes a tumour‐specific promoter (hTERTp, human telomerase reverse transcriptase) and the promoters of the *E1A* gene (necessary for viral replication), cytomegalovirus (CMV) and promoters that can activate the apoptin gene (*Apoptin*). Therefore, Ad‐VT is a dual‐specific oncolytic adenovirus that has the ability to specifically kill tumour cells and exerts tumour‐specific replication capability. Ad‐T (Ad‐hTERTp‐E1A), Ad‐VP3 (Ad‐CMV‐Apoptin) and Ad‐Mock are all viruses that can be used as controls. These four recombinant oncolytic adenoviruses have been confirmed in previous studies to have strong inhibitory effects on the growth of lung cancer, prostate cancer and liver cancer.^8^, ^9^

Bioluminescent imaging in vivo is a highly useful visualization technique that can be used to track cells and analyse tissue activity and gene behaviour in vivo.^10^ Bioluminescence imaging in vivo is characterized by light scattering and is associated with a unique imaging advantage^11^; since there is almost no endogenous luminescence in tissues and cells, the endogenous signal‐to‐noise ratio is low; therefore, background interference can be effectively eliminated, and the bioluminescence signal can be clearly observed in complex organisms.

The establishment of a luciferase‐labelled animal tumour model would provide intuitive, real‐time and continuous monitoring of the growth and metastasis of different tumour models with the aid of a live animal imaging system. This type of system could detect very slight changes over time, thus providing an ideal animal model for the more intuitive evaluation of the efficacy of anticancer drugs. In this study, we aimed to construct a luciferase‐labelled human breast cancer cell line that could more intuitively and continuously detect the effect of anticancer drugs on the inhibition of tumour growth in vivo.

In this study, we investigated the inhibitory effect of recombinant oncolytic adenovirus Ad‐VT on two human breast cancer cell lines (MCF‐7 and MDA‐MB‐231). The characteristics of recombinant oncolytic adenovirus therapy, traditional chemotherapy and endocrine therapy were also compared. Using luciferase‐labelled human breast cancer lines (MCF‐7 Luc and MDA‐MB‐231 Luc), we established a luciferase‐labelled tumour model in nude mice and further explored the feasibility of gene therapy for breast cancer. Our intention was to provide preliminary evidence for the future application of combinations of oncolytic adenoviruses with chemotherapy drugs for the treatment of breast cancer.

## 2 MATERIALS AND METHODS

### 2.1 Human breast cancer cell lines, oncolytic adenovirus, drugs and animals

Two human breast cancer cell lines (MCF‐7 and MD‐MB‐231) were purchased from the Cell Bank of the Shanghai Institute for Biological Science (Shanghai, China). Frozen normal mammary epithelial MCF‐10A cells were obtained from the Changchun Veterinary Research Institute. MCF‐7 cells were cultured in RPMI 1640 basic medium containing 10% foetal bovine serum, 50 U/ml penicillin and 50 U/ml streptomycin at 37°C with 5% CO_2_. MDA‐MB‐231 cells were cultured in DMEM basic medium containing10% foetal bovine serum, 50 U/ml penicillin and 50 U/ml streptomycin medium at 37°C with 5% CO_2_. MCF‐10A cells were cultured in DMEM basic medium containing 10% foetal bovine serum, 100 U/ml penicillin and 100 U/ml streptomycin at 37°C with 5% CO_2_. Foetal bovine serum, RPMI 1640 basic medium and DMEM basic medium were purchased from Thermo Fisher Scientific. Penicillin and streptomycin were purchased from HyClone GE Healthcare Life Sciences. The four recombinant adenoviruses (MOCK, Ad‐VP3, Ad‐T and Ad‐VT) were provided by our group. Doxorubicin and tamoxifen were purchased from Sigma‐Aldrich, USA. Doxorubicin stock solution (10 mg/ml) was diluted with culture medium to concentrations of 50 μg/ml (86 μmol/L), 5 μg/ml (8.6 μmol/L), 0.5 μg/ml (0.86 μmol/L) and 0.05 μg/ml (0.086 μmol/L). Tamoxifen stock solution (10 mmol/L) was diluted with culture medium to concentrations of 25, 20, 15 and 10 μmol/L. Female nude mice aged 4–5 weeks were purchased from Beijing Vital River Laboratory Animal Technology Co. Ltd. The animal experimental protocols were approved by the Institutional Animal Care and Use Committee of the Changchun University of Chinese Medicine. The cages for rearing SPF grade BALB/c female nude mice were cleaned, sterilized and high pressured. We used SPF grade sterilized rat chow and litter, and autoclaved the water given to the nude mice. The rearing environment was a sterile, constant temperature and humidity animal room, and the animals were reared adaptively for 10 days before tumour bearing.

### 2.2 Measurement of cell viability

#### 2.2.1 WST‐1 assays

**FIGURE 2:**
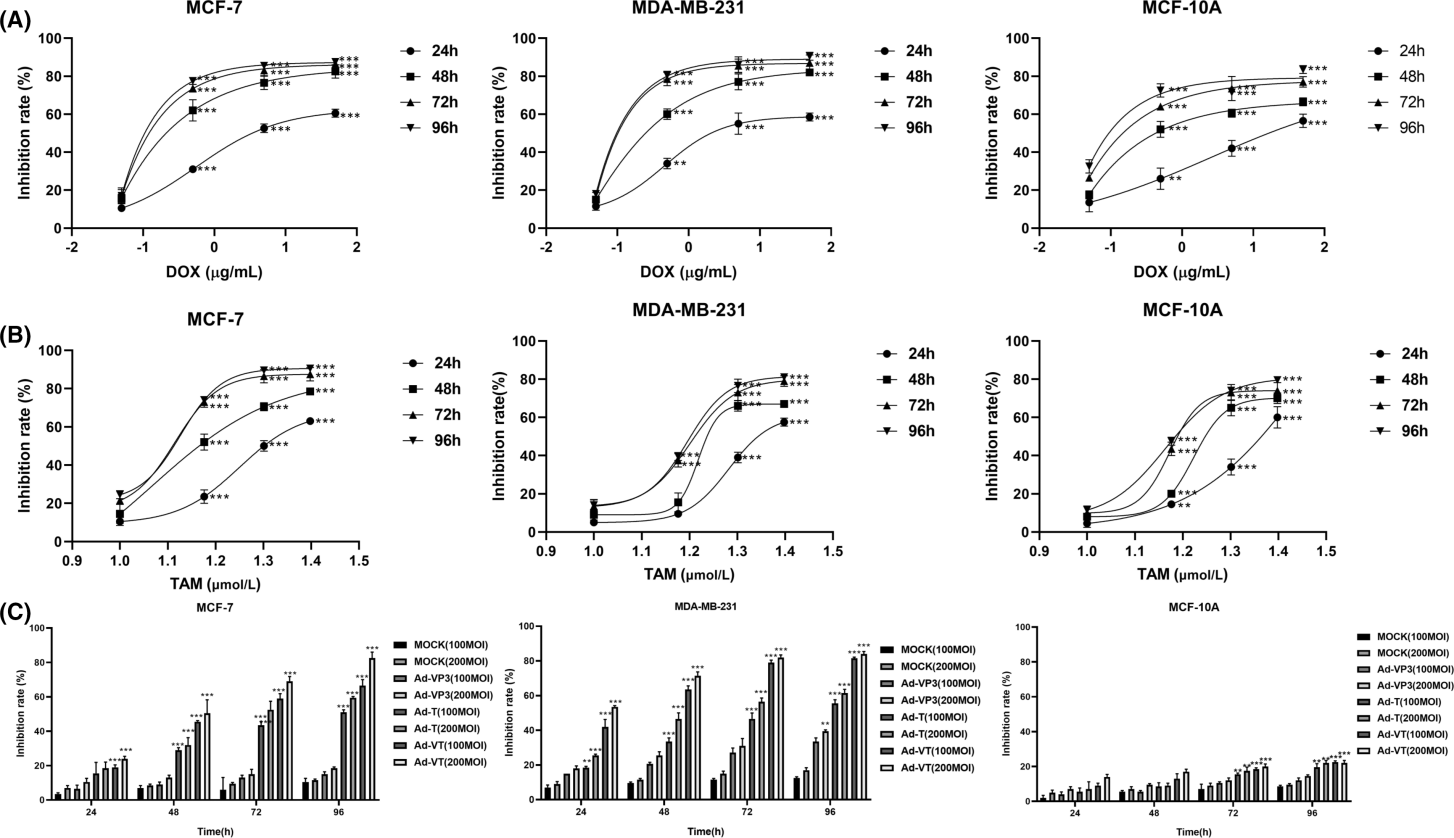
Effects of doxorubicin, tamoxifen and recombinant oncolytic adenovirus on the viability of MCF‐7, MDA‐MB‐231 and MCF‐10A cells, as determined by WST‐1 assays. The three cell types (MCF‐7, MDA‐MB‐231 and MCF‐10A) were treated with doxorubicin (0.05, 0.5, 5 and 50 μg/ml) (A), tamoxifen (10, 15, 20 and 25 μmol/L) (B), and four recombinant oncolytic adenoviruses (MOCK, Ad‐VP3, Ad‐T and Ad‐VT) (C). Then, we tested viability in each group after 24, 48, 72 and 96 h. All measurements were performed in triplicate, and the mean ± standard deviation was compared with the 0.05 μg/ml, 10 μmol/L and MOCK (100moi and 200moi) group (*p < 0.05, **p < 0.01, ***p < 0.001)

MCF‐7, MDA‐MB‐231 and MCF‐10A cells undergoing logarithmic growth were cultured in a 96‐well plate at a density of 5 × 10^3^ cells/well with 100 μl of culture medium per well. Cells were cultured for 24 h in a 5% CO_2_ incubator; then, the three cell types were infected with four recombinant oncolytic adenoviruses (MOCK, Ad‐VP3, Ad‐T and Ad‐VT) at 100 and 200 MOI, respectively. Two other 96‐well plates, with the same cell density, were also prepared. The cells in these plates received doxorubicin (0.05, 0.5, 5 and 50 μg/ml) and tamoxifen (10, 15, 20 and 25 μmol/L). Each group was reproduced in triplicate. The cells (including those undergoing viral infection and drug administration) were cultured in a 5% CO_2_ incubator at 37°C for 24, 48, 72 and 96 h. Prior to detection, the cell proliferation reagent WST‐1 (Sigma‐Aldrich, USA) was added to each well; cells were then cultured in the dark for 90 min in a 5% CO_2_ incubator at 37°C. Control wells were treated in the same manner. The absorbance value of each well was measured at 490 nm using an Infinite 200 multifunctional microplate reader (Tecan Trading AG, Switzerland) (Figure 2), and the cell proliferation inhibition rate was calculated according to the following formula: cell proliferation inhibition ratio (%) = (control well absorbance value A‐treated well absorbance value A)/control well absorbance value A × 100%.

### 2.3 Detection of apoptosis

#### 2.3.1 Hoechst staining

The slides were placed in a 6‐well plate to prepare monolayers of MCF‐7 and MDA‐MB‐231 cells at a cell density of 2.5 × 10^5^ cells/well. MCF‐7 and MDA‐MB‐231 cells were then infected with 100 MOI of recombinant oncolytic adenovirus (MOCK, Ad‐VP3, Ad‐T and Ad‐VT). We also prepared 6‐well plates containing the same cell density for the administration of doxorubicin (0.05, 0.5 and 5 μg/ml) and tamoxifen (10, 15 and 20 μmol/L) for 24, 48 and 72 h. At the end of incubation, the culture medium was discarded and 1 ml of Hoechst (Life Technologies, USA) staining solution (diluted to 1:1000) was added to each well. Cells were then cultured in the dark in a 5% CO_2_ incubator at 37°C for 8 min. After incubation, the working solution was discarded, and the cells were washed twice with PBS. Culture medium was then added, and the slides were analysed by fluorescence microscopy (BX‐60, Olympus, Tokyo, Japan) (excitation filter wavelength: 355 nm; block filter wavelength: 512 nm), and photographed.

#### 2.3.2 Annexin V‐FITC/PI staining

Monolayers of MCF‐7 and MDA‐MB‐231 cells were prepared in 6‐well plates at a cell density of 2.5 × 10^5^ cells/well and then cultured at 37°C in a 5% CO_2_ incubator for 24 h. Then, the cells were infected with 100 MOI of recombinant oncolytic adenovirus (MOCK, Ad‐VP3, Ad‐T or Ad‐VT). We also prepared 6‐well plates with the same cell density to administer doxorubicin (0.05, 0.5 and 5 μg/ml) and tamoxifen (10, 15 and 20 μmol/L). The cells were treated with drugs and recombinant oncolytic adenovirus for 24, 48 and 72 h. MCF‐7 and MDA‐MB‐231 cells were then treated with the FITC‐coupled Annexin‐V Apoptotic Kit (Cell Quest Pro, Becton Dickinson) for 24, 48 and 72 h, and the levels of apoptosis were detected by FACS flow cytometry (C6 Plus and FACSCalibur, Becton Dickinson, Franklin Lakes, NJ, USA).

### 2.4 Mitochondrial membrane potential and the detection of apoptotic proteins

#### 2.4.1 JC‐1 staining

Slides were placed in a 6‐well plate to prepare monolayers of MCF‐7 and MDA‐MB‐231 cells at a cell density of 2.5 × 10^5^ cells/well. The cells were then infected with 100 MOI of recombinant oncolytic adenovirus (MOCK, Ad‐VP3, Ad‐T or Ad‐VT). We also prepared 6‐well plates with the same cell density to administer doxorubicin (0.05, 0.5 and 5 μg/ml) and tamoxifen (10, 15 and 20 μmol/L). The cells were then treated with drugs and recombinant oncolytic adenovirus for 24, 48 and 72 h. Once the experiment was complete, we discarded the medium and washed the cells twice with PBS. Then, we added 1 ml of JC‐1 (Life Technologies, USA) staining solution (diluted 1:1000 dilution of JC‐1 staining solution 5 ng/ml) and incubated for 15 min in the dark at 37°C in a 5% CO_2_ incubator. Next, the working solution was discarded, and the cells were washed twice with PBS. Cells were then analysed by fluorescence microscopy (BX‐60, Olympus, Tokyo, Japan) (excitation filter wavelength: 485 nm; blocking filter wavelength: 530 nm; excitation filter wavelength: 530 nm; and blocking filter wavelength: 590 nm), and representative images were acquired.

MCF‐7 cells and MDA‐MB‐231 cells that were in the logarithmic growth phase were cultured in a 96‐well plate at a density of 5 × 10^3^ cells/well with a culture volume of 100 μl per well; these were incubated for 24 h at 37°C in a 5% CO_2_ incubator. The cells were then infected with 100 MOI of recombinant oncolytic adenovirus (MOCK, Ad‐VP3, Ad‐T or Ad‐VT). We also prepared 6‐well plates with the same cell density to administer doxorubicin (0.05, 0.5 and 5 μg/ml) and tamoxifen (10, 15 and 20 μmol/L); each group was set up in triplicate. At the end of the observation period, we discarded the medium, washed the cells twice with PBS and then added 100 μl of JC‐1 (Life Technologies, USA) staining solution (1:1000 dilution of JC‐1 staining solution 5 ng/ml). Then, we incubated the cells for 15 min in the dark at 37°C in a 5% CO_2_ incubator. Next, we discarded the working solution, wash the cells twice with PBS and then added serum‐ and antibiotic‐free RPMI 1640 and DMEM medium. The results were then determined by an Infinite 200 multifunctional microplate reader (Tecan Trading AG, Switzerland).

### 2.5 The detection of apoptotic proteins

Suspensions of MCF‐7 and MAD‐MB‐231 cells were seeded in a 6‐well cell culture plate at a density of 3 × 10^5^ cells/well. Cells were then infected with 100 MOI of MOCK, Ad‐VP3, Ad‐T or Ad‐VT. After 48 h, we used Western blotting to determine the levels of key proteins in the two types of cells; to do this, we incubated the cells with antibodies raised against PARP, cleaved Caspase‐3 and cytochrome C (Cell Signalling Technology, USA).

After 48 h of infection, the cells were harvested by centrifuged at 2400 *g* for 5 minutes. Then, 200 μl of cell lysis reagent SD‐001 from the Minute™ Total Protein Extraction Kit (Invent, Germany) was added to the cell pellets. Cell lysates were then homogenized by repeated pipetting and then centrifuged at 18500 *g* in a precooled centrifuge tube sleeve for 30 s (4°C). The final concentration of protein extracts was then determined using a BCA Protein Quantification Kit (Beyotime Biotechnology, China). Western blotting was then used to analyse the levels of the three proteins related to apoptosis.

After 48 h of infection, the cells were harvested by centrifugation at 2400 *g* for 5 min (4°C). Mitochondria were then isolated with a Minute™ Mitochondrial Isolation Kit (Invent, Germany). Mitochondrial protein was then extracted by IP lysis buffer (Beyotime Biotechnology, China). The concentrations of the extracted proteins were then determined with a BCA Protein Quantification Kit (Beyotime Biotechnology, China). Western blotting was then used to determine the protein levels of cytochrome C in each of the extracts.

### 2.6 Generation of luciferase‐labelled human breast cancer cells

Human breast cancer cells (MCF‐7 and MDA‐MB‐231) undergoing the logarithmic growth phase were seeded onto a 6‐well plate. When the cells reached 80% confluence, they were transfected with the pGL4.51 plasmid (Promega Corporation, USA). Twenty‐four hours after transfection, a single‐cell suspension containing 400 μg/ml of G418 (Geneticin) (BD Bioscience Clontech, San Diego, CA, USA) was prepared and seeded into a new 6‐well plate for extended culture. The media (G418) was refreshed every 2–3 days until monoclonal cells were detected. We selected monoclonal cells with good tolerance from the cell culture plates and continued to grow these cells in 24‐well plates in G418 medium. When the degree of fusion was >90%, the cells were transferred into 96‐well plates for culture and were then assayed for luciferase activity. Cell lines with the highest clonal luminescence value were inoculated on a 96‐well plate, and different concentration gradients were generated. After 24 h, we added thee luciferase substrate (Promega Corporation, USA) and measured bioluminescence intensity with a small animal live imaging system (Merck KGaA, Darmstadt, Germany).

### 2.7 Determination of the cell cycle before and after construction of the cell lines

Suspensions of MCF‐7 and MCF‐7 Luc cells were prepared at a cell density of 1.5 × 10^5^ cells/ml and added to a 6‐well plate containing 2 ml per well. After 24 h of cell culture, the cells were collected in a centrifuge tube and centrifuged at 400 *g* for 5 min. The supernatant was discarded, and the cells were washed in PBS. Then, the cells were precooled with 75% ethanol, mixed well and cultured in the dark at 4°C for 24 h. Next, the samples were washed in PBS and then staining solution before another centrifugation step at 400 *g* for 10 min. The supernatant was then discarded, and PI/RNase (Becton Dickinson, USA) staining solution was added to resuspend the cells; the cells were then stirred in the dark for 15 min. Next, we determined the cell cycle stage of the samples by flow cytometry (FACSCalibur, Becton Dickinson, Franklin Lakes, NJ, USA). The method used to determine the cell cycle in MDA‐MB‐231 and MDA‐MB‐231 Luc cells was the same as that for MCF‐7 and MCF‐7 Luc cells.

### 2.8 Determination of growth curves before and after cell line construction

MCF‐7 and MCF‐7 Luc cell suspensions were prepared and added to a 96‐well plate and cultured. The 96‐well cell culture plates were removed from culture at seven different time points (day one to day seven); at each point, the medium in each well was discarded and Cell Proliferation Reagent WST‐1 (Sigma‐Aldrich, USA) (110 μl of a 1:10 dilution of WST‐1) was added. The plates were then placed at 37°C in a 5% CO_2_ incubator and incubated in the dark for 2 h. After shaking for 10s, the OD value of the cells at 450 nm was then measured with an Infinite 200 multifunctional microplate reader (Tecan Trading AG, Switzerland). The growth curve of MDA‐MB‐231 and MDA‐MB‐231 Luc cells was determined by the same method.

### 2.9 Animal models

MCF‐7 Luc cells (cell density 1 × 10^7^/100 μl) and MDA‐MB‐231 Luc cells (cell density 1 × 10^7^/100 μl) were injected subcutaneously into the right hind limb of BALB/C nude mice. After the tumour‐bearing model was established, the mice were randomly divided into five groups according to the type of tumour‐bearing cells: a MOCK treatment group, an Ad‐VP3 treatment group, an Ad‐T treatment group, an Ad‐VT treatment group and a control group (10 mice per group). The purified recombinant adenovirus (1 × 10^9^ TCID50/100 μl) was injected every 3 days for 3 weeks. Before detecting tumour size, the nude mice were injected intraperitoneally with luciferin (Promega Corporation, USA) at a dose of 200 μl/mouse (15 mg/ml). After 5 min, each mouse was intraperitoneally injected with 1% sodium pentobarbital (120 μl) for anaesthesia. Photographs of the tumour sites were taken once a week for 5 weeks to observe changes in survival time, and the luminescence intensity of tumours was detected by a small animal live imaging system (Merck KGaA, Darmstadt, Germany). Five weeks after the photographs were taken, nude mice were euthanized with excess CO_2_, and the livers and kidneys of the non‐model group (the nude mice without subcutaneous tumour, three nude mice) and Ad‐VT groups (three nude mice) were removed and HE staining was performed to analyse whether there were changes in the structural state of the organs and whether there was an inflammatory reaction. The MCF‐7 Luc cell tumour‐bearing group and the MDA‐MB‐231 Luc cell tumour‐bearing group received the same treatment and analysis methods.

### 2.10 Statistical processing

Statistical analyses were conducted using data from at least three independent experiments, and GraphPad Prism version 7 (GraphPad Software, USA) was used to process the data in the form of ($\bar{x}\pm s$). Comparisons between the two groups were analysed by the *t*‐test. Comparisons were significant when *p* < 0.05 or *p* < 0.01.

## 3 RESULTS

### 3.1 Growth inhibition of breast cancer cells and mammary epithelial cells by a recombinant oncolytic adenovirus and two drugs (doxorubicin and tamoxifen)

WST‐1 was used to detect and analyse the inhibitory effect of recombinant oncolytic adenovirus and two clinical drugs (doxorubicin and tamoxifen) on the proliferation of breast cancer cells and normal breast epithelial cells.

Doxorubicin not only significantly inhibited the proliferation of breast cancer cells (MCF‐7 and MDA‐MB‐231) and normal breast epithelial cells (MCF‐10A) (*p* < 0.01). The inhibitory rate increased with as the drug concentration increased. As shown in Figure 2A, doxorubicin inhibited the proliferation of the three cell types at four time points. After 48 h of doxorubicin treatment, the inhibitory rates of doxorubicin (0.5 μg/ml) on the three cell types (MCF‐7, MDA‐MB‐231 and MCF‐10A) were 58%, 62% and 55%, respectively. As time and dose increased, the inhibitory rate also increased; the inhibitory effect was similar for the three types of cells. When the drug concentration was 50 μg/ml, the inhibitory rate for the three cell types exceeded 80% after 96 h.

The effect of tamoxifen on MCF‐7, MDA‐MB‐231 and MCF‐10A cells is shown in Figure 2B. At the four time points, the three drug concentrations inhibited the three cell types to varying degrees. The inhibitory rate increased as the drug concentration increased. When the drug concentration was 15 μmol/L, the inhibitory rates of tamoxifen on MCF‐7 cells were 21% at 24 h, 43% at 48 h, 75% at 72 h and 75% at 96 h. The inhibitory rates of tamoxifen on MDA‐MB‐231 cells were 10% at 24 h, 19% at 48 h, 40% at 72 h and 41% at 96 h. The inhibitory rates of tamoxifen on MCF‐10A cells were 14% at 24 h, 19% at 48 h, 41% at 72 h and 46% at 96 h. After 72 h and at a dose of 25 μmol/L, tamoxifen exerted an inhibitory effect of more than 70% in the three cell types.

The effect of Ad‐VT is shown in Figure 2C. Ad‐VT specifically inhibited the proliferation of MCF‐7 and MDA‐MB‐231 tumour cells but had no obvious inhibitory effect on normal breast epithelial cell. The inhibitory rate of recombinant oncolytic adenovirus on the two forms of breast cancers was ranked as follows: Ad ‐ VT > Ad‐T > Ad ‐ VP3 > MOCK (*p* < 0.01). At the same dose of infection, the inhibitory rate of Ad‐VT on breast cancer cells increased significantly as the infection time increased. The inhibition rate of the four recombinant oncolytic adenoviruses on the three cells was greater than 100MOI when the infection dose was 200MOI. We also identified a time‐ and dose‐dependent relationship. After 72 h, the inhibitory rate of Ad‐VT (100MOI) on normal mammary epithelial cells was <20%, while the inhibitory rate on the two types of breast cancer cells (MCF: 61%; MDA‐MB‐231: 78%) exceeded 60%, thus significantly inhibiting the proliferation of breast cancer cells. After 96 h, the inhibition rate of Ad‐VT (200MOI) on normal breast epithelial cells was 23%, while the inhibition rate on two breast cancer cells was MCF: 80%; MDA‐MB‐231: 83%. When the concentration of doxorubicin is 50 and 5 μg/ml, the inhibition rate of the two breast cancer cells was essentially the same (both around 80%). The WST‐1 results showed that the inhibition rate of doxorubicin after 72 and 96 h was essentially the same; the S curve became stable, and the inhibition rate did not increase significantly. The inhibitory effect of doxorubicin on cells at 96 h was essentially the same as that at 72 h; when the concentration of tamoxifen was 20 μmol/L, the S curve became stable, and the inhibition rate did not increase significantly when the concentration of tamoxifen increased. When the infection dose of oncolytic adenovirus was 200MOI and 100MOI, the mortality rate of cells was essentially the same, and the inhibition rate did not increase significantly after 72 h. Therefore, in our subsequent experiments, the drug concentrations of tamoxifen were selected as 20, 15 and 10 μmol/L, and the drug concentrations of doxorubicin were selected as 5, 0.5 and 0.05 μg/ml; the dose of oncolytic adenovirus was selected at 100 MOI, and the time points of detection were 24, 48 and 72 h.

Collectively, WST‐1 staining showed that Ad‐VT had the most significant inhibitory effect on the proliferation of tumour cells but had almost no inhibitory effect on normal breast epithelial cells. In contrast, doxorubicin not only had a strong inhibitory effect on breast cancer cells but also inhibited the proliferation of normal breast epithelial cells. Tamoxifen only had a strong inhibitory effect on oestrogen‐dependent breast cancer cells, had no obvious inhibitory effect on triple‐negative breast cancer, but also had a strong inhibitory effect on normal breast epithelial cells.

### 3.2 Recombinant oncolytic adenovirus and two drugs (doxorubicin and tamoxifen) increased apoptosis in breast cancer cells

**FIGURE 3:**
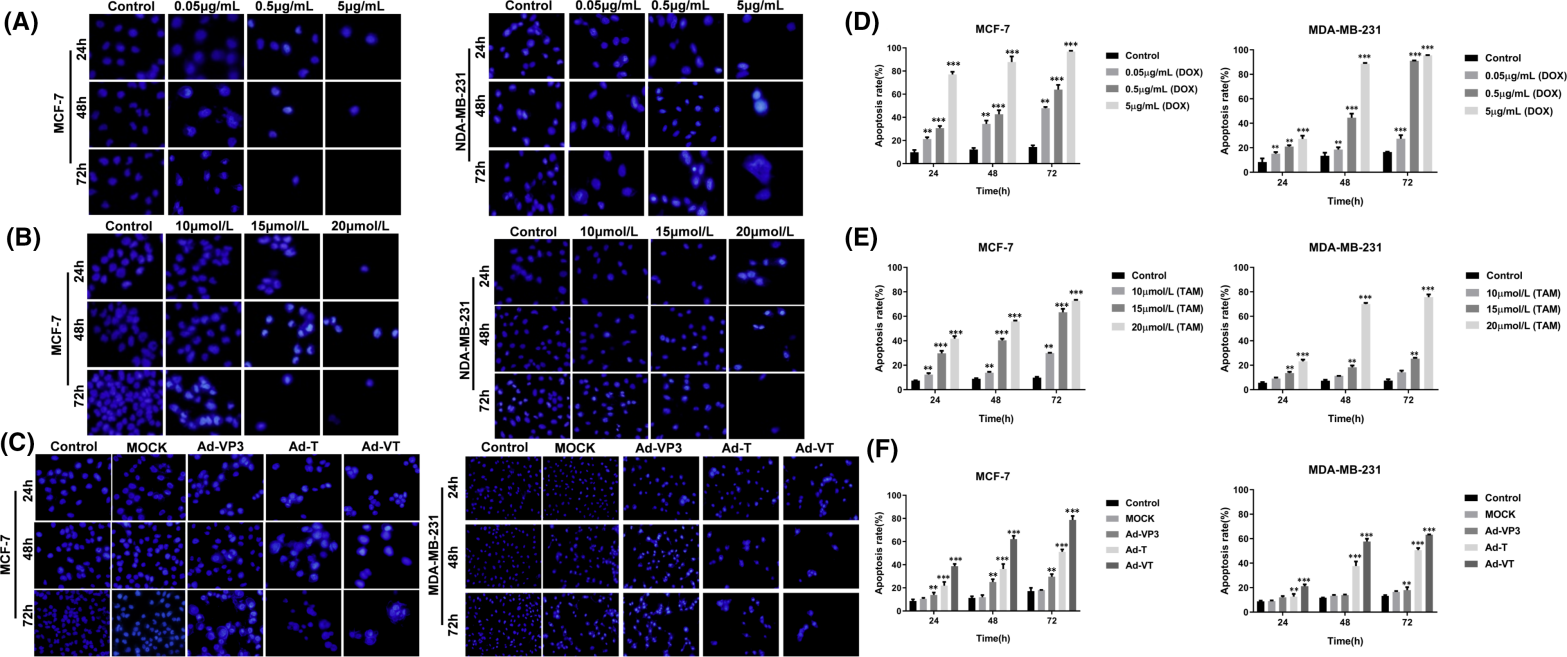
Apoptosis of MCF‐7 and MDA‐MB‐231 cells induced by doxorubicin, tamoxifen and recombinant oncolytic adenovirus, as detected by Hoechst staining and Annexin V flow cytometry. Two breast cancer cells (MCF‐7 and MDA‐MB‐231) were treated with different concentrations of doxorubicin (A), tamoxifen (B) and recombinant oncolytic adenovirus (C). Morphological changes of breast cancer cell nuclei were observed by Hoechst staining at 24, 48 and 72 h. Two breast cancer cell types (MCF‐7 and MDA‐MB‐231) were treated with doxorubicin (D), tamoxifen (E) and recombinant oncolytic adenovirus (F). The extent of apoptosis in breast cancer cells was analysed by flow cytometry using Annexin‐V FITC/PI dye at 24, 48 and 72 h. All measurements were performed in triplicate, and the mean ± standard deviation was compared to the control group (*p < 0.05, **p < 0.01, ***p < 0.001)

Hoechst is a class of dye that can enter cells passively and bind to nucleic acids to exhibit blue fluorescence. As shown in Figure 3A, when the concentration of doxorubicin was 0.5 and 5 μg/ml, the two types of breast cancer cells were significantly reduced at different time points; cell nuclei exhibited obvious and bright nuclear staining with evidence of nuclear fragmentation. The degree of bright staining was positively correlated with the prolongation of time and an increase in drug concentration.

Tamoxifen‐treated MCF‐7 and MDA‐MB‐231 cells did not exhibit bright nuclear staining at a concentration of 10 μmol/L. When the concentration of the drug was 15 μmol/L, the nuclei of MCF‐7 cells exhibited strong nuclear staining and decreased cell number, while MDA‐MB‐231 cells exhibited uniform blue fluorescence with no nuclear fragmentation. When the drug concentration was 20 μmol/L, the nuclei of both breast cancer cell types exhibited strong nuclear fluorescence and reduced the number of cells (Figure 3B). After MCF‐7 and MDA‐MB‐231 cells were treated with the four recombinant adenoviruses (Ad‐VT, Ad‐T, AdVP3 and MOCK), some of the cell nuclei in the Ad‐VT, Ad‐T and Ad‐VP3 groups exhibited strong blue fluorescence. The lowest number of cells was evident in the Ad‐VT group and there as obvious nuclear fluorescence and fragmentation in this group. The MOCK and control groups exhibited blue fluorescence, and the cells were denser (Figure 3C).

Annexin V‐FITC/PI flow cytometry assays showed that doxorubicin had a strong effect on the induction of apoptosis in both breast cancer cell types (MCF‐7 and MDA‐MB‐231); the rate of apoptosis increased significantly with increasing drug dose and time (*p* < 0.01) (Figure 3D). When the drug concentration exceeded 0.5 μg/ml, there was evidence of extensive apoptosis; the rate of apoptosis gradually increased with both administration time and drug concentration. The rate of cellular apoptosis exceeded 40% after 48 h of administration.

There was significant difference in the rates of apoptosis when compared between MCF‐7 and MDA‐MB‐231 cells treated with tamoxifen. When the drug concentration was 15 μmol/L, after 24 h of drug treatment, the apoptosis rate in MCF‐7 cells was 31% while that in MDA‐MB‐231 cells was just 12%. After 48 h, the rate of apoptosis in MCF‐7 cells was 41% while that in MDA‐MB231 cells was 17%. After 72 h, the rate of apoptosis in MCF‐7 cells was 61% while that of MDA‐MB‐231 cells was 24%. However, when the drug concentration was 20 μmol/L, both two breast cancer cell types exhibited strong apoptosis; the rate of apoptosis was similar when compared between the two cell types (Figure 3E).

The four recombinant adenoviruses all induced apoptosis in two types of breast cancer cells (MCF‐7 and MDA‐MB‐231) at three different time points; however, the extent of apoptosis differed. As shown in Figure 3F, the extent of apoptosis in breast cancer cells induced by recombinant oncolytic adenovirus at different time points followed a specific trend: Ad‐VT > Ad‐T > Ad‐VP3 > MOCK. Ad‐VT induced the highest rate of apoptosis in tumour cells in which the rate of apoptosis was >60% after 72 h of infection. Furthermore, the induction in breast cancer cells by Ad‐VT exhibited a significant time/effect relationship (*p* < 0.01).

Hoechst and Annexin V‐FITC/PI staining further showed that doxorubicin induced apoptosis in breast cancer cells and that the rate of apoptosis increased gradually with time and dose. Tamoxifen also induced apoptosis in oestrogen‐dependent breast cancer cells (MCF‐7) but only at a concentration of 15 μmol/L. Tamoxifen induced a lower degree of apoptosis in triple‐negative breast cancer MDA‐MB‐231 cells. Collectively, data showed that the four recombinant oncolytic adenoviruses inhibited cancer cell proliferation by inducing apoptosis in breast cancer cells and that Ad‐VT had the strongest ability to induce apoptosis in cancer cells.

### 3.3 Recombinant oncolytic adenovirus and two drugs (doxorubicin and tamoxifen) induced apoptosis in breast cancer cells by altering the mitochondrial membrane potential

**FIGURE 4:**
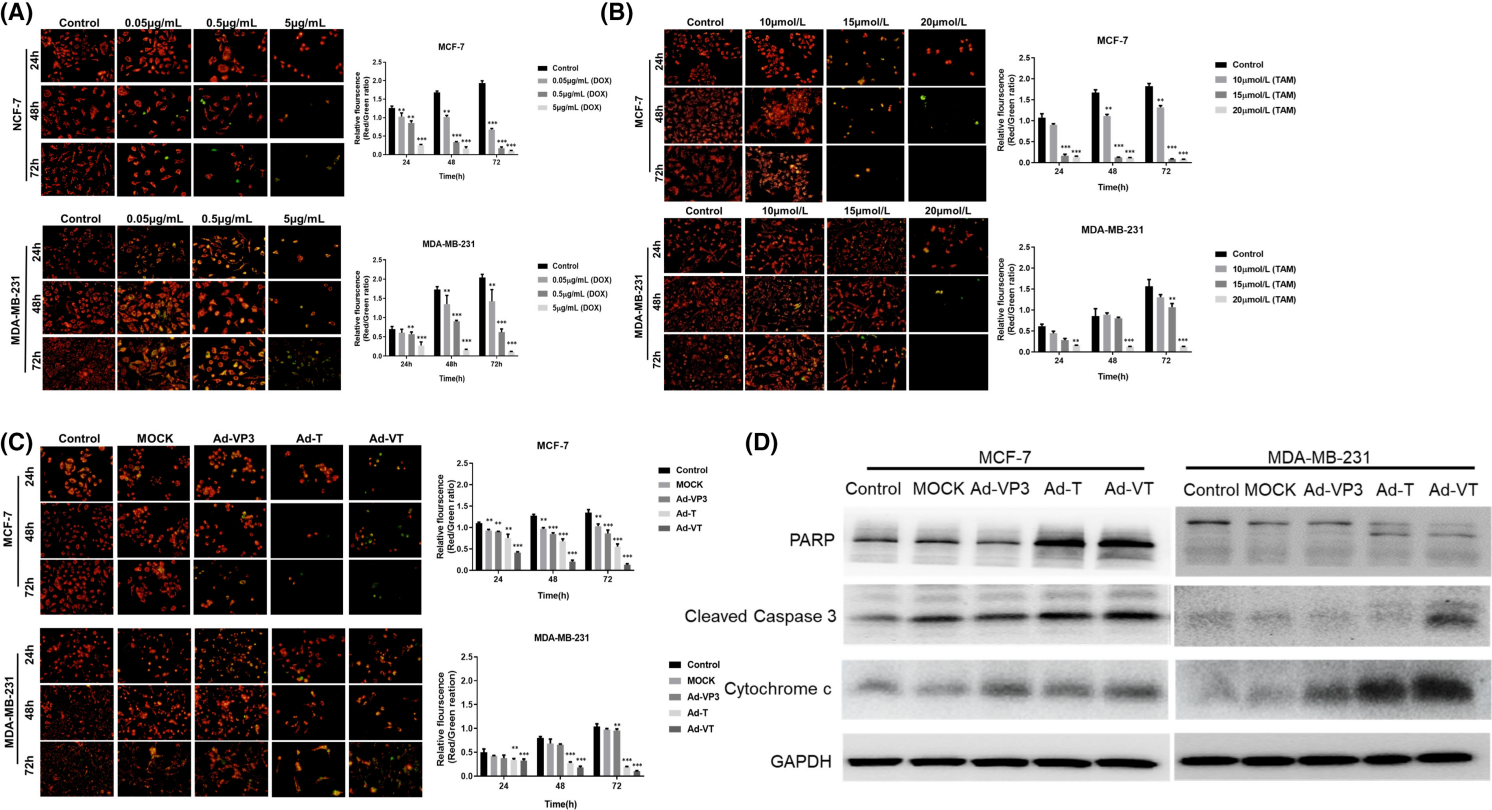
JC‐1 staining was used to analyse changes in the mitochondrial membrane potential of breast cancer cells induced by doxorubicin, tamoxifen and recombinant oncolytic adenovirus. We also used Western blotting to detect the expression of PARP, cleaved caspase‐3 and cytochrome C protein in breast cancer cells in response to recombinant oncolytic adenovirus. Two types of breast cancer cells (MCF‐7 and MDA‐MB‐231) were treated with different concentrations of doxorubicin (A), tamoxifen (B) and recombinant oncolytic adenovirus (C). Changes in the mitochondrial membrane potential of breast cancer cells were observed by JC‐1 staining at 24, 48 and 72 h. MCF‐7 and MDA‐MB‐231 cells were infected with four recombinant adenolytic adenoviruses. After 48 h, we extracted total protein proteins from the cells and used Western blotting to detect the protein expression levels of PARP, caspase‐3 and cytochrome C (D). All measurements were performed in triplicate; the mean ± standard deviation were then compared with the control group (*p < 0.05, **p < 0.01, ***p < 0.001)

Doxorubicin was used to treat MCF‐7 and MDDA‐MB‐231 breast cancer cells, and JC‐1 staining solution was added at 24, 48 and 72 h for analysis. As shown in Figure 4A, doxorubicin induced significant changes in the mitochondrial membrane potential of two breast cancer cell types; the trend for change in mitochondrial potential was essentially the same when compared between the two cell types. The ratio of red fluorescence/green fluorescence for mitochondrial membrane potential followed a specific trend: Control > 0.05 > 0.5 > 5 μg/ml (*p* < 0.01). For example, at a concentration of 0.5 μg/ml, 48 h after administration, the number of cells decreased significantly; furthermore, the number of cells exhibiting red fluorescence gradually decreased while the number exhibiting green fluorescence gradually increased. The ratio of red to green fluorescence decreased as administration time and drug concentration increased.

Tamoxifen was used to treat MCF‐7 and MDDA‐MB‐231 breast cancer cells and induced apoptosis in both types of breast cancer cells by changing their mitochondrial membrane potential, as shown in Figure 4B. When the concentration of tamoxifen was 10 μmol/L, the mitochondrial membrane potential of the two types of breast cancer cells did not change significantly and the rate of apoptosis was low. However, at a dose of 15 μmol/L, the extent of apoptosis in MCF‐7 cells that was induced by tamoxifen was significant (*p* < 0.01); fluorescence microscopy further showed that red fluorescent aggregates had gradually shifted to green fluorescent monomers. After 72 h of treatment, the number of apoptotic MCF‐7 cells was significantly higher than that in MDA‐MB‐231 cells, and the ratio of red fluorescence to green fluorescence had reduced significantly (*p* < 0.01). At 20 μmol/L, tamoxifen induced extensive levels of apoptosis in the two breast cancer cell types; the mitochondrial membrane potential was also significantly reduced at all three time points; the ratio of red fluorescence to green fluorescence was as follows: Control < 10 μmol/L < 15 μmol/L < 20 μmol/L (*p* < 0.01).

Recombinant oncolytic adenovirus was used to infect both MCF‐7 and MDA‐MB‐231 breast cancer cells. Infection with oncolytic adenovirus induced apoptosis in breast cancer cells to varying degrees at the three observation points (Figure 4C). Prolonged infection of breast cancer cells by recombinant oncolytic adenovirus enhanced the ability of Ad‐VT and Ad‐T to induce changes in the membrane potential of cancer cells; this led to an increase in the number of apoptotic cells. The number of breast cancer cells was lowest after 72 h of infection; at this point, there was a significant change in the mitochondrial membrane potential of the cancer cells (*p* < 0.05). At the three specific time points, the ratio of red fluorescence/green fluorescence followed a certain sequence: Ad‐VT < Ad‐T < Ad‐VP3 < MOCK.

MCF‐7 and MDA‐MB‐231 cells were infected with four recombinant adeno‐oncolytic adenoviruses. After 48 h, we extracted total protein from both cells and mitochondria and analysed these proteins by Western blotting (Figure 4D). We found that Ad‐VT induced the cleavage of PARP and Caspase‐3 in breast cancer cells and promoted the release of mitochondrial cytochrome C. This suggested that Ad‐VT can induce apoptosis in breast cancer cells by changing their mitochondrial membrane potential.

To summarize, JC‐1 staining showed that doxorubicin could induce apoptosis in breast cancer cells by inducing changes in their mitochondrial membrane potential. As the administration time and dosage increased, the mitochondrial membrane potential of cancer cells gradually decreased. At a concentration of 15 μmol/L, tamoxifen induced strong mitochondrial membrane potential changes in oestrogen‐dependent breast cancer MCF‐7 cells; Western blotting further showed that Ad‐VT induced significant apoptosis in breast cancer cells by changing their mitochondrial membrane.

### 3.4 Construction of luciferase‐labelled human breast cancer cells

**FIGURE 5:**
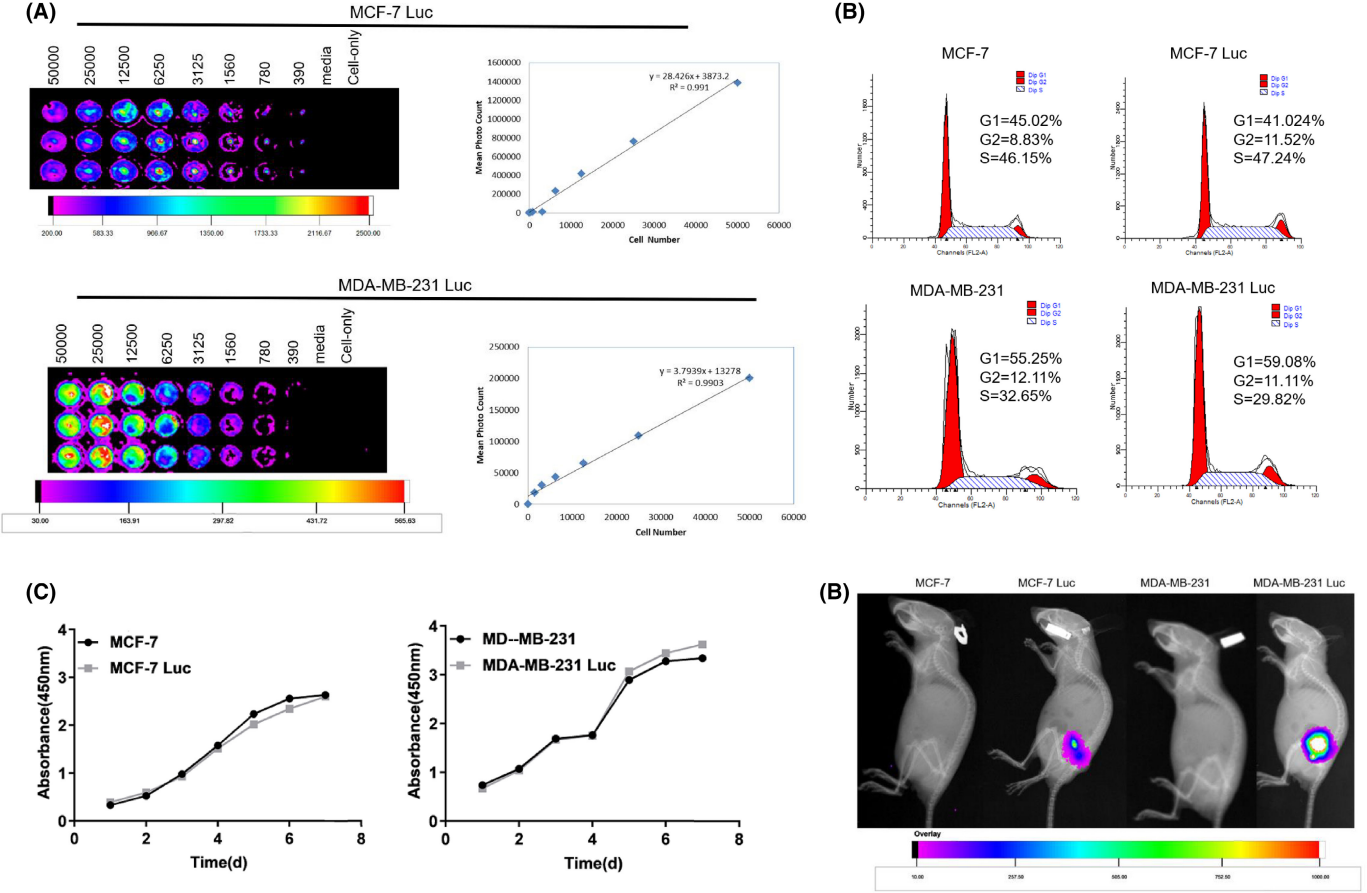
Construction and characterization of two luciferase‐labelled breast cancer cell lines (MCF‐7 Luc and MDA‐MB‐231 Luc). (A) MCF‐7 Luc and MDA‐MB‐231 Luc were inoculated into 96‐well plates according to the principle of multiple dilution. After adding luciferase, the luminescence intensity was observed. The luminescence intensity increased as the number of cells increased. (B) MCF‐7, MCF‐7 Luc, MDA‐MB‐231 and MDA‐MB‐231 Luc cells were seeded and cultured in a 6‐well cell plate, and the cell cycle was detected by flow cytometry. (C) MCF‐7, MCF‐7 Luc, MDA‐MB‐231 and MDA‐MB‐231 Luc cells were cultured on a 96‐well cell plate. Cell growth rates were detected on Days 1, 2, 3, 4, 5, 6 and 7. (D) MCF‐7, MCF‐7 Luc, MDA‐MB‐231 and MDA‐MB‐231 Luc were inoculated subcutaneously in the right hind limbs of nude mice at a cell density of 1 × 10^7^. Subsequently, we determined the luminescence intensity of the tumours formed in the nude mice

As shown in Figure 5A, two luciferase‐labelled cell lines (MCF‐7 Luc and MDA‐MB‐231 Luc) were constructed. The luminescence values of these labelled cell lines exhibited a linear relationship with the number of cells; the more cells, the higher the luminescence value.

Figure 5B shows results relating to cell cycle determination. Before and after the construction of the two cell types, the proportion of cells in each phase was essentially the same (G1 phase, G2 phase and S phase) and there were no significant changes between the cell types with regard to the cell cycle (*p* > 0.05). Growth curves for the two cell types are shown in Figure 5C; these curves were similar before and after construction indicating that the growth characteristics of the two cell types were not significantly different (*p* > 0.05).

Figure 5D shows the data arising from in vivo imaging experiments; breast cancer cells (MCF‐7 Luc and MDA‐MB‐231 Luc) exhibited strong luminescence in the inoculum area, while other types of breast cancer cells (MCF‐7 and MDA‐MB‐231) did not exhibit any luminescence in the inoculum area.

In summary, cell growth curves and cell cycle experiments showed that there were no significant changes between the two cell types in terms of their biological characteristics before and after construction. Luciferase activity assays, along with in vivo tumour formation experiments in nude mice, further confirmed that the two luciferase‐labelled human breast cancer cell lines expressed luciferase in a stable manner. Collectively, these results indicated that the stable expression of luciferase did not affect the growth characteristics of cells.

### 3.5 In vivo imaging of subcutaneous tumours in BALB/c nude mice and survival analysis

**FIGURE 6:**
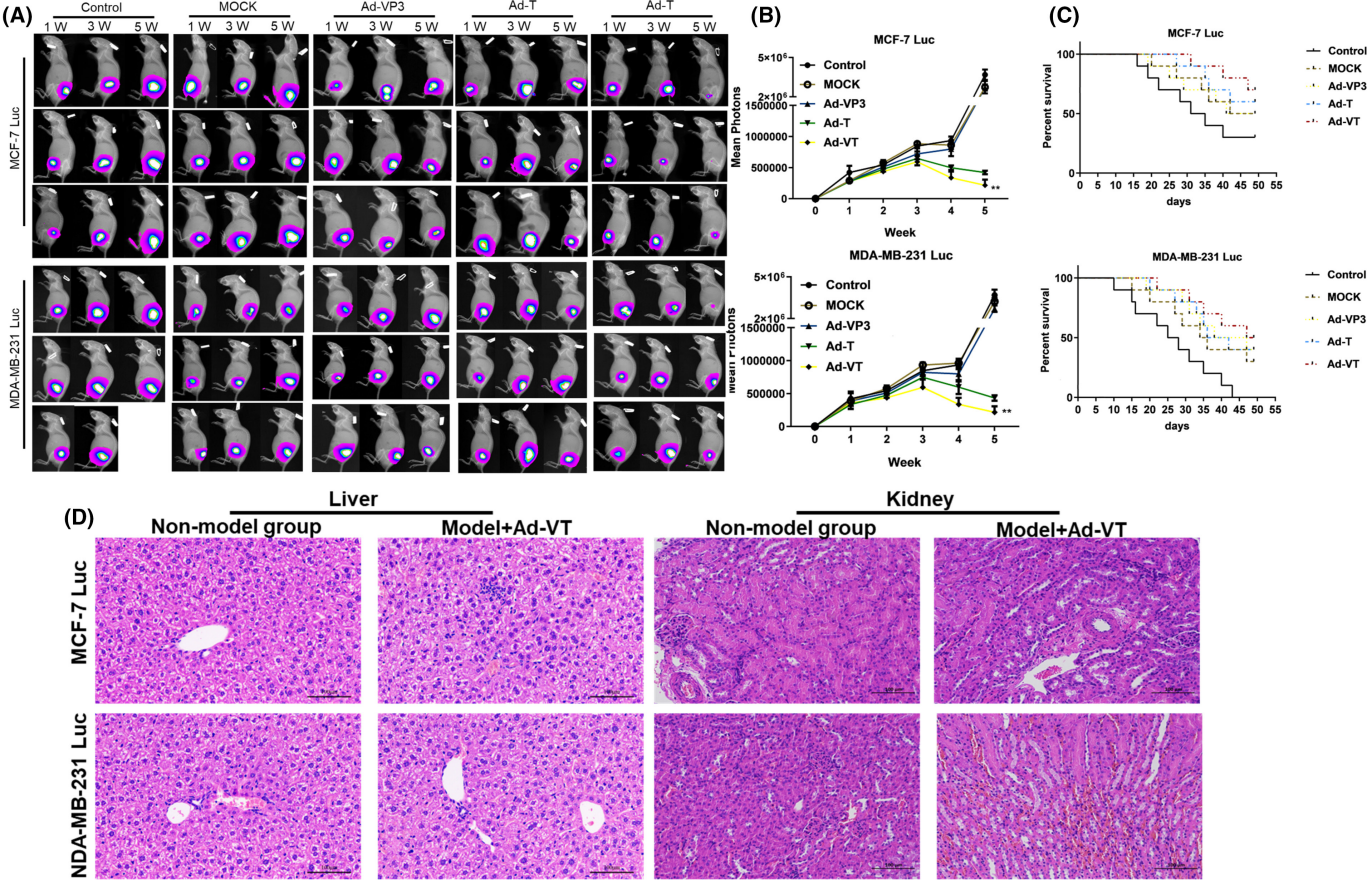
Effects of recombinant oncolytic adenovirus on breast cancer in a BALB/c nude mice model. (A, B) MCF‐7 Luc and MDA‐MB‐231 Luc cells (1 × 10^7^/100 μl) were injected subcutaneously into the right hind limb of nude mice (10 mice in each group) to establish a xenograft model. An in vivo imaging luminescence system was used to continuously monitor changes in tumour bioluminescence intensity. (C) After successfully establishing the xenograft model in nude mice, we recorded the survival rates of mice daily for 5 weeks. The mean tumour inhibition rate in the 1 × 10^9^ PFU/100 μl Ad‐VT treatment group was significantly higher than that of the other groups. The survival rate of the 1 × 10^9^ PFU/100 μl Ad‐VT treatment group was also the highest of all groups; the mean survival rate of nude mice exceeded 50%. The survival time of mice in the 1 × 10^9^ PFU/100 μl Ad‐VT treatment group was significantly longer than those in the control group or the 1 × 10^9^ PFU/100 μl MOCK treatment group. (D) After 5 weeks of continuous photography, the livers and kidneys of nude mice in the non‐model group and the Ad‐VT treatment group were taken for HE staining (three mice in each group). (*p < 0.05, **p < 0.01, ***p < 0.001)

The bioluminescence intensity of tumours in nude mice was continuously detected with a small animal imaging system for 5 weeks from Week 0. The bioluminescence results are shown in Figure 6A,B. After 1–3 weeks of treatment with four recombinant oncolytic adenoviruses, there was no significant difference in the mean bioluminescence intensity of the tumour area when compared between nude mice bearing MCF‐7 Luc cells and MDA‐MB‐231 Luc cells (*p* > 0.05). During Weeks 3–4, the bioluminescence intensity of the tumours in the Ad‐VT and Ad‐T treatment groups increased slowly when compared with the control group. During Weeks 4–5 weeks, the bioluminescence intensity of the tumour area in the Ad‐VT treatment group was significantly lower than that in the control group (*p* < 0.01); the levels of luminescence from tumour cells followed a specific sequence: Ad‐VT < Ad‐T < Ad‐VP3 < MOCK < Control. We used excess CO_2_ to euthanize nude mice at the end of the experiment. The livers and kidneys of the non‐model group (nude mice without subcutaneous tumour) and Ad‐VT treatment groups were removed for HE staining. As shown in Figure 6D, the liver tissue structure of the nude mice in the Ad‐VT treatment group was normal, and the hepatocytes were arranged in an orderly manner without obvious change; the kidney tissue structure of nude mice after intra‐tumoral injection of Ad‐VT was normal, the size of glomeruli was relatively uniform, the structure of renal tubules was normal, and there was no obvious degeneration of epithelial cells, intra‐tumoral injection of Ad‐VT treatment group and nude mice without subcutaneous tumour had no changes in tissue sections, and the morphology was similar. Ad‐VT had no effect on the liver and kidney of nude mice. Following the development of tumours, we recorded the survival of nude mice in each group. Compared with the other treatment groups (Ad‐VP3, Ad‐T and Ad‐VT), the nude mice in the MOCK treatment group had all died by Day 10 after subcutaneous tumour loading; the mean survival time of mice in this group was significantly shorter than that in other groups (Figure 6C). The mean survival rate of nude mice in the Ad‐VT treatment group exceeded 50%; in addition, the total survival period was significantly prolonged in this group.

Nude mice treated with Ad‐T and Ad‐VT were still alive 50 days after the formation of tumours; the survival rate of mice in the Ad‐VT treatment group was higher (*p* < 0.01) than that of mice in the Ad‐T treatment group.

## 4 DISCUSSION

Cancer is one of the most significant and dangerous health issues in the world today and can seriously affect the health and lives of those affected. Over recent years, the global incidence of breast cancer has increased; this particular cancer is now the most common global cancer^12^; however, the molecular mechanisms underlying breast cancer have yet to be elucidated.^13^

There are many different treatment strategies for patients with breast cancer, including surgery, radiation, chemistry, endocrine therapy and HER2 molecular‐targeted therapy. Surgery and chemotherapy are still the main clinical treatment methods, although these are often associated with significant side effects after chemotherapy. These side effects can seriously affect the quality of life of patients. Endocrine therapy and HER2 molecular‐targeted therapy can effectively alleviate the symptoms experienced by most patients, although there are many limitations associated with these therapeutic options. The effects of these two treatments are not satisfactory for patients with cancers that are the most difficult to treat such as those with triple‐negative breast cancer (MDA‐MB‐231).^14^, ^15^, ^16^

Over recent years, there has been significant development in biomedicine and genetic engineering technology; consequently, many new treatment options have been developed, including immunotherapy and gene therapy. Oncolytic viruses are viruses that can selectively infect and replicate in tumour cells.^17^, ^18^ These viruses can kill a variety of tumour cells and have low levels of toxicity.^19^, ^20^

The focus of oncolytic adenovirus therapy refers to the use of a virus‐mediated immune response to inhibit tumour progression. During the process of oncolytic adenovirus treatment, the host immune system will eventually completely clear the virus, a process that requires that the virus used in the treatment of cancer is safe for the host. Ideally, the adenovirus can induce an anti‐tumour immune response before it is cleared. Compared with other types of tumour treatment, oncolytic viruses are effective for a variety of tumours, have low toxicity, and cost‐effective with regard to manufacturing costs.

Apoptin was the first tumour‐selective anti‐oncogene to be isolated and is derived from the apoptosis‐inducing protein of chicken anaemia virus. Chicken anaemia virus features three open reading frames that encode three proteins VP1 (capsid protein: 51.6 kDa), VP2 (scaffold protein: 24.0 kDa) and VP3 (apoptosis‐inducing protein: 13.6 kDa). The protein expressed by VP3 is referred to as ‘apoptin’ because it can induce apoptosis in tumour cells^21^, ^22^ but has no effect on normal diploid cells in humans. Telomerase is mainly responsible for the de novo synthesis of nucleotides that are used to extend the ends of chromosomes that are found in germ cells and most malignant cells. Human telomerase reverse transcriptase (hTERTp) acts as a specific promoter in tumour cells and can trigger the replication or expression of certain genes (e.g. *E1A* and *Apoptin*) in tumour cells.^23^, ^24^

The establishment of luciferase‐labelled animal tumour models provides us with the ability to monitor the proliferation and metastasis of cells in different tumour models in real time, thus allowing us to detect subtle physiological changes over time in small animals.

By performing a series of validation experiments, we found that when expressing the *Luc* gene, breast cancer cells exhibited similar growth characteristics as normal breast cancer cells; the basic growth characteristics of the cells did not change significantly. In vitro imaging experiments further showed that there was a linear relationship between the fluorescent intensity of MCF‐7 Luc and MDA‐MB‐231 Luc and the number of cells; the correlation coefficient was >0.99. Therefore, in animal experiments, MCF‐7 Luc can replace MCF‐7 cells, and MDA‐MB‐231 Luc can replace MDA‐MB‐231 cells to establish useful tumour models; subsequently, growth and metastasis can be investigated by the application of in vivo imaging systems. These findings provide a cytological foundation for the development of exciting new tumour models.

A previous study by Zhang et al. in 2020 showed that when compared with cisplatin, a new oncolytic adenovirus (Ad‐TD‐nsIL12), carrying human non‐secretory IL‐12, significantly inhibited tumour cell growth and tumour angiogenesis and improved the survival rate of experimental animals but without any notable side effects.^25^ The recombinant adenovirus described in the present study can also kill breast cancer cells. WST‐1 results showed that Ad‐VP3, Ad‐T and Ad‐VT all caused significant mortality in breast cancer cells; the inhibitory rates produced by these viruses followed a specific sequence: Ad‐VP3 < Ad‐T < Ad‐VT. Furthermore, these viruses had only minimal inhibitory effects on mammary epithelial cells. After 72 h, the inhibitory rate of Ad‐VT on mammary epithelial cells was <20%. The chemotherapeutic drug doxorubicin not only inhibited the proliferation of breast cancer cells; it also induced a high inhibitory rate in normal breast epithelial cells. When the concentration of doxorubicin was 5 μg/ml, the inhibitory rate on MCF‐10A cells was >70% at the 72‐h timepoint. The endocrine drug tamoxifen only exerted a high inhibitory rate (75%) on oestrogen‐dependent cells (MCF‐7); this drug had a much lower inhibitory rate (40%) on triple‐negative breast cancer cells (MDA‐MB‐231). We also used Hoechst and Annexin V‐FITC/PI staining to investigate the pathways by which the recombinant adenoviruses were able to kill breast cancer cells. We found that Ad‐VT, Ad‐T and Ad‐VP3 all induced apoptosis in mammary cells and were able to kill these cells; the rate of apoptosis generated followed a specific sequence: Ad‐VT > Ad‐T > Ad‐VP3. Doxorubicin induced significant apoptosis in two types of breast cancer cells; as the drug concentration and the period of administration increased, the rate of apoptosis in the two types of cancer cells also increased; the rates of apoptosis followed a specific trend: Control < 0.05 < 0.5 < 5 μg/ml. When the concentration of tamoxifen was 15 μmol/L, the extent of apoptosis in oestrogen‐dependent MCF‐7 cells increased significantly after 72 h; the rate of apoptosis was >60%, and the number of cells had reduced significantly. Apoptosis was not extensive in MDA‐ MB‐231 cells; the specific rate of apoptosis was <30%. JC‐1 staining and Western blotting experiments showed that the recombinant adenoviruses (Ad‐VT, Ad‐T and Ad‐VP3) all induced cell apoptosis by changing the mitochondrial membrane potential. We found that Ad‐VT induced the cleavage of PARP and Caspase‐3 in two breast cancer cells, and released cytochrome C in the mitochondria of cancer cells, thus inducing extensive endogenous apoptosis in cancer cells.

A recent study by Mao et al.,^26^ involving an animal model used a combination of oncolytic adenovirus ZD55‐SATB1 to target SATB1 and DTX treatment to effectively inhibit the growth of xenograft tumours; this effect was accompanied by an increase in the expression of caspase protein and a reduction in the expression of CD31. In addition, Xiao et al. showed that the oncolytic adenovirus CD55‐Smad 4 effectively inhibited the proliferation of colorectal cancer cells both in vivo and in vitro, while also activating the caspase signal transduction pathway and inducing apoptosis in colorectal cancer cells.^27^ In the present study, we found that the mean luminescent intensity of tumours in the control group was significantly higher than that in any of the other treatment groups. During Weeks 3–5, the mean luminescent intensity of the Ad‐VT treatment group was lower than that produced by the other treatment groups. We also found that the survival rate of mice in the recombinant oncolytic adenovirus treatment group was significantly prolonged (the mean survival rate of nude mice in the Ad‐VT treatment group exceeded 50%). Collectively, these results indicate that Ad‐VT and Ad‐T significantly inhibited tumour growth in vivo and improved the survival rate of experimental mice.

## 5 CONCLUSION

Our research demonstrated that although the chemotherapeutic drug doxorubicin can kill tumour cells very effectively, it also caused the death of normal breast epithelial cells. Tamoxifen only had a strong inhibitory effect on oestrogen‐dependent breast cancer cells and had no obvious inhibitory effect on triple‐negative breast cancer, but also had a strong inhibitory effect on normal breast epithelial cells. Compared with these two forms of first‐line chemotherapy drugs (doxorubicin and tamoxifen), we found that Ad‐VT had a significant inhibitory effect on both types of breast cancer cells and induced apoptosis and death in breast cancer cells by changing their mitochondrial membrane potential. The inhibitory rate of Ad‐VT on mammary epithelial cells was only minimal. In vivo experiments further showed that Ad‐VT effectively inhibited the growth of tumour cells and prolonged the survival time of treated mice. This discovery provides a new direction for the treatment of breast cancer and the possibility of administering combined treatments featuring oncolytic adenoviruses, chemotherapeutic drugs and endocrine drugs.

## AUTHOR CONTRIBUTIONS

**Shanzhi Li:** Data curation (supporting); formal analysis (lead); writing – original draft (lead); writing – review and editing (supporting). **Zhuoxin Li:** Data curation (supporting); software (supporting). **Yiquan Li:** Methodology (supporting); software (supporting). **Yilong Zhu:** Data curation (supporting); software (supporting). **Jicheng Han:** Formal analysis (supporting). **Wenjie Li:** Data curation (supporting). **Ningyi Jin:** Formal analysis (supporting). **Jinbo Fang:** Software (lead); writing – original draft (lead). **Guangze Zhu:** Data curation (lead); funding acquisition (lead); writing – original draft (supporting).

## CONFLICT OF INTEREST

There is no conflict of interest in this article.

## Data Availability

The data that support the findings of this study are available from the corresponding author upon reasonable request.

## References

[1] Elhawary S , El‐Hefnawy H , Mokhtar FA , et al. Jasminum officinal green synthesis of silver nanoparticles using extract of L. Leaves and evaluation of cytotoxic activity towards Bladder (5637) and breast cancer (MCF‐7) cell lines. Int J Nanomedicine. 2020;15:9771‐9781. doi:10.2147/IJN.S269880 33304101 PMC7723236

[2] Tzekaki EE , Geromichalos G , Lavrentiadou SN , Tsantarliotou MP , Pantazaki AA , Papaspyropoulos A . Oleuropein is a natural inhibitor of PAI‐1‐mediated proliferation in human ER‐/PR‐ breast cancer cells. Breast Cancer Res Treat. 2021;186(2):305‐316. doi:10.1007/s10549-020-06054-x 33389400

[3] Davey MG , Ryan ÉJ , McAnena PF , et al. Disease recurrence and oncological outcome of patients treated surgically with curative intent for estrogen receptor positive, lymph node negative breast cancer. Surg Oncol. 2021;37:101531. doi:10.1016/j.suronc.2021.101531 33545657

[4] Wan BA , Pidduck W , Zhang L , et al. Patient‐Reported Pain in Patients with Breast Cancer Who Receive Radiotherapy. Pain Manag Nurs. 2021;22(3):402‐407. doi:10.1016/j.pmn.2020.12.007 33485785

[5] Carbine NE , Lostumbo L , Wallace J , Ko H . Risk‐reducing mastectomy for the prevention of primary breast cancer. Cochrane Database Syst Rev. 2018;4(4):CD002748. doi:10.1002/14651858.CD002748.pub4 29620792 PMC6494635

[6] Jiang J , Cole D , Westwood N , et al. Crucial roles for protein kinase C isoforms in tumor‐specific killing by apoptin. Cancer Res. 2010;70(18):7242‐7252. doi:10.1158/0008-5472 20719884

[7] Malla WA , Arora R , Khan RIN , Mahajan S , Tiwari AK . Apoptin as a tumor‐specific therapeutic agent: current perspective on mechanism of action and delivery systems. Front Cell Dev Biol. 2020;8:524. doi:10.3389/fcell.2020.00524 32671070 PMC7330108

[8] Jin J , Zhu YL , Sun F , Chen ZF , Chen S , et al. Synergistic antitumor effect of the combination of a dual cancer‐specific oncolytic adenovirus and cisplatin on lung cancer cells. Oncol Lett. 2018;16:6275‐6282. doi:10.3892/ol.2018.9470 30405762 PMC6202551

[9] Li YQ , Zhu YL , Fang JB , et al. Apoptin regulates apoptosis and autophagy by modulating reactive oxygen species (ROS) levels in human liver cancer cells. Front Oncol. 2020;10:1026. doi:10.2289/fonc.2020.01026 32714864 PMC7344208

[10] Dou L , Matz EL , Gu X , et al. Non‐Invasive Cell Tracking with Brighter and Red‐Transferred Luciferase for Potential Application in Stem Cell Therapy. Cell Transplant. 2019;28(12):1542‐1551. doi:10.1177/0963689719885078 31684762 PMC6923553

[11] Rahman SU , Stanton M , Casey PG , et al. Listeria monocytogenes development of a click beetle luciferase reporter system for enhanced bioluminescence imaging of: analysis in cell culture and murine infection models. Front Microbiol. 2017;8:1797. doi:10.3389/fmicb.2017.01797 29018414 PMC5622934

[12] Hotton J , Lusque A , Leufflen L , et al. Early locoregional breast surgery and survival in de novo metastatic breast cancer in the multicenter national ESME cohort. Ann Surg. 2021. doi:10.1097/SLA.0000000000004767. 33534229

[13] Nichol A , Narinesingh D , Raman S , et al. The effect of bolus on local control for patients treated with mastectomy and radiotherapy. Int J Radiat Oncol Biol Phys. 2021;110(5):1360‐1369. doi:10.1016/j.ijrobp.2021.01.019 33485893

[14] Wang YH , Chen YH , Shen WH . Amikacin suppresses human breast cancer cell MDA‐MB‐231 migration and invasion. Toxics. 2020;8(4):108. doi:10.3390/toxics8040108 PMC7712503 33233497

[15] Zhang Z , Lu M , Chen C , et al. Holo‐lactoferrin: the link between ferroptosis and radiotherapy in triple‐negative breast cancer. Theranostics. 2021;11(7):3167‐3182. doi:10.7150/thno.52028 33537080 PMC7847686

[16] El‐Aziz YSA , Spillane AJ , Jansson PJ , Sahni S . Role of ABCB1 in mediating chemo‐resistance of triple negative breast cancers. Biosci Rep. 2021;41(2):BSR20204092. doi:10.1042/BSR20204092 33543229 PMC7909869

[17] Atasheva S , Emerson CC , Yao J , Young C , Stewart PL , Shayakhmetov DM . Systemic cancer therapy with engineered adenovirus that evades innate immunity. Sci Transl Med. 2020;12(571):eabc6659. doi:10.1126/scitranslmed.abc6659 33239388 PMC9195642

[18] Gao J , Zhang W , Ehrhardt A . Expanding the spectrum of adenoviral vectors for cancer therapy. Cancer. 2020;12(5):1139. doi:10.3390/cancers12051139 PMC7281331 32370135

[19] Santos JM , Heiniö C , Cervera‐Carrascon V , et al. Oncolytic adenovirus shapes the ovarian tumor microenvironment for potent tumor‐infiltrating lymphocyte tumor reactivity. J Immunother Cancer. 2020;8(1):e000188. doi:10.1136/jitc-2019-000188 31940588 PMC7057530

[20] Xiao B , Zhang L , Liu H , et al. Oncolytic adenovirus CD55‐Smad4 suppresses cell proliferation, metastasis, and tumor stemness in colorectal cancer by regulating Wnt/β‐Catenin signaling pathway. Biomedicine. 2020;8(12):593. doi:10.3390/biomedicines8120593 PMC7763845 33322272

[21] Alam MM , Kariya R , Boonnate P , Kawaguchi A , Okada S . Induction of apoptosis by Shikonin through ROS‐mediated intrinsic and extrinsic apoptotic pathways in primary effusion lymphoma. Transl Oncol. 2021;14(3):101006. doi:10.1016/j.tranon.2020.101006 33401054 PMC7785961

[22] Fu Y , Ye Y , Zhu G , et al. Resveratrol induces human colorectal cancer cell apoptosis by activating the mitochondrial pathway via increasing reactive oxygen species. Mol Med Rep. 2021;23(3):170. doi:10.3892/mmr.2020.11809 33398363

[23] Ke W , Zhao X , Lu Z . Foeniculum vulgare seed extract induces apoptosis in lung cancer cells partly through the down‐regulation of Bcl‐2. Biomed Pharmacother. 2021;135:111213. doi:10.1016/j.biopha.2020.111213 33395604

[24] Zingue S , Cisilotto J , Fogang RCM , et al. The antimammary tumor effects of ethanolic extract of propolis from Adamawa region (Cameroon) are by apoptosis via reactive oxygen species‐mediated mitochondrial pathway. Environ Toxicol. 2021;36(5):861‐873. doi:10.1002/tox.23089 33393727

[25] Zhang Z , Zhang C , Miao J , et al. A tumor‐targeted replicating oncolytic adenovirus Ad‐TD‐nsIL12 as a promising therapeutic agent for human esophageal squamous cell carcinoma. Cell. 2020;9(11):2438. doi:10.3390/cells9112438 PMC7698064 33182528

[26] Mao L , Yu H , Ma S , et al. Combination of oncolytic adenovirus targeting SATB1 and docetaxel for the treatment of castration‐resistant prostate cancer. J Cancer. 2021;12(6):1846‐1852. doi:10.7150/jca.46868 33613773 PMC7890306

[27] Xiao B , Zhang L , Liu H , et al. Oncolytic adenovirus CD55‐Smad4 suppresses cell proliferation, metastasis, and tumor stemness in colorectal cancer by regulating Wnt/beta‐catenin signaling pathway. Biomedicine. 2020;8(12):593. doi:10.3390/biomedicines8120593 PMC7763845 33322272

